# The impact of co-infections on the haematological profile of East African Short-horn Zebu calves

**DOI:** 10.1017/S0031182013001625

**Published:** 2013-10-25

**Authors:** ILANA CONRADIE VAN WYK, AMELIA GODDARD, B. MARK DE C. BRONSVOORT, JACOBUS A. W. COETZER, IAN G. HANDEL, OLIVIER HANOTTE, AMY JENNINGS, MAIA LESOSKY, HENRY KIARA, SAM M. THUMBI, PHIL TOYE, MARK W. WOOLHOUSE, BANIE L. PENZHORN

**Affiliations:** 1 Department of Veterinary Tropical Diseases, Faculty of Veterinary Science, University of Pretoria, Private bag X04, Onderstepoort, 0110, South Africa; 2 Clinical Pathology, Department Companion Animal Medicine, Faculty of Veterinary Science, University of Pretoria, Private bag X04, Onderstepoort, 0110, South Africa; 3 The Roslin Institute at the R (D) SVS, University of Edinburgh, Easter Bush, EH25 9RG, UK; 4 School of Biology, University of Nottingham, Nottingham NG7 2RD, UK; 5 Department of Production Animal Health, Faculty of Veterinary Science, University of Pretoria, Private bag X04, Onderstepoort, 0110, South Africa; 6 Department of Medicine, University of Cape Town, 1000, South Africa; 7 International Livestock Research Institute, P.O. Box 30709-00100, Nairobi, Kenya; 8 Centre for Immunology, Infection and Evolution, University of Edinburgh, EH9 3JT, UK

**Keywords:** co-infections, packed cell volume, white blood cell count, platelets, Zebu, calves

## Abstract

The cumulative effect of co-infections between pathogen pairs on the haematological response of East African Short-horn Zebu calves is described. Using a longitudinal study design a stratified clustered random sample of newborn calves were recruited into the Infectious Diseases of East African Livestock (IDEAL) study and monitored at 5-weekly intervals until 51 weeks of age. At each visit samples were collected and analysed to determine the infection status of each calf as well as their haematological response. The haematological parameters investigated included packed cell volume (PCV), white blood cell count (WBC) and platelet count (Plt). The pathogens of interest included tick-borne protozoa and rickettsias, trypanosomes and intestinal parasites. Generalized additive mixed-effect models were used to model the infectious status of pathogens against each haematological parameter, including significant interactions between pathogens. These models were further used to predict the cumulative effect of co-infecting pathogen pairs on each haematological parameter. The most significant decrease in PCV was found with co-infections of trypanosomes and strongyles. Strongyle infections also resulted in a significant decrease in WBC at a high infectious load. Trypanosomes were the major cause of thrombocytopenia. Platelet counts were also affected by interactions between tick-borne pathogens. Interactions between concomitant pathogens were found to complicate the prognosis and clinical presentation of infected calves and should be taken into consideration in any study that investigates disease under field conditions.

## INTRODUCTION

Infectious diseases are a major constraint to livestock production in East Africa (Perry and Young, 1995). The tropical climate in western Kenya is conducive to the survival of many infectious pathogens and vectors. The economically most important diseases of livestock in sub-Saharan Africa are tick-borne diseases, especially East Coast fever (ECF), heartwater, anaplasmosis and babesiosis, and also trypanosomosis (Uilenberg, 1995; Minjauw and McLeod, 2003; Maudlin, 2006) and helminthosis. Small-holder farmers are particularly vulnerable to the economic impact of infectious diseases on livestock. Losses include lowered production rates, mortalities, decreased reproduction rates and costs of treatment and control measures. These diseases also indirectly constrain livestock production through limiting the use of the highly susceptible improved breeds of livestock that are used in other countries to improve livestock productivity (Perry and Young, 1995).

Animals living under natural conditions are more likely to suffer from multi-pathogen infectious burdens than single infections (Cox, 2001; Telfer *et al*. 2008). The higher the prevalence of each pathogen, the more likely an individual host will harbour co-infections of the various pathogens (Petney and Andrews, 1998). Swai *et al*. (2005) reported that cattle in Tanzania that were seropositive to one tick-borne pathogen were more likely to be seropositive to another tick-borne pathogen. Several studies have reported concomitant infections of trypanosomes, *Anaplasma, Babesia, Theileria* and helminth species in cattle in Africa (Magona and Mayende, 2002; Swai *et al*. 2005; Kamani *et al*. 2010; Marufu *et al*. 2010), and particularly Kenya (Moll *et al*. 1984; Maloo *et al*. 2001; Muraguri *et al.* 2005).

It is easy to conceive that pathogens that concomitantly infect a host would directly interact with each other, particularly pathogens that occupy the same niche in the host, e.g. the abomasum or red blood cells. These pathogens often compete for the same resources, such as nutrients, attachment sites, etc. The result of direct competition is often a limit on population size for either or both of the implicated pathogens (Petney and Andrews, 1998). Pathogens can also interact indirectly by modifying the host's immunity against (Holmes *et al*. 1974; Mackenzie *et al.* 1975; Urquhart and Holmes, 1987; Kaufmann *et al.* 1992; Lachhman *et al*. 2010; Tabel *et al*. 2013) or susceptibility to other infections (Holmes *et al*. 1974; Mackenzie *et al*. 1975). Interactions between co-infecting pathogens can further alter the course of the resultant infection, for example, by reduced or prolonged prepatent periods (Kaufmann *et al*. 1992; Gale *et al.* 1997), or increased pathogenicity of pathogens (Kaufmann *et al*. 1992; Goossens *et al.* 1997; Petney and Andrews, 1998). It is thus more relevant to study infectious disease in the context of the complete pathogenic neighbourhood of the host since each pathogen ultimately contributes to the clinical outcome and prognosis of infection in the individual host.

In this study the impact of co-infections of specified pathogens on the haematological response of East African Short-horn Zebu calves was investigated. Newborn calves were recruited into a longitudinal study in which each calf was monitored and sampled at 5-weekly intervals. The infectious status of each calf at each sampling point was modelled against specific haematological responses, including packed cell volume (PCV), total white blood cell count (WBC) and platelet count (Plt). The pathogens of interest included tick-borne pathogens (*Theileria parva, Theileria mutans, Anaplasma marginale* and *Babesia bigemina*), trypanosomes and intestinal parasites (strongyle- and strongyloides type nematodes, coccidia and fasciola). The models also investigated the presence of significant interactions between pathogens. Predicted outcomes of these models were computed to illustrate the cumulative effect of co-infections between different pathogen pairs on each haematological parameter.

## MATERIALS AND METHODS

### Study design

**Fig. 1.**
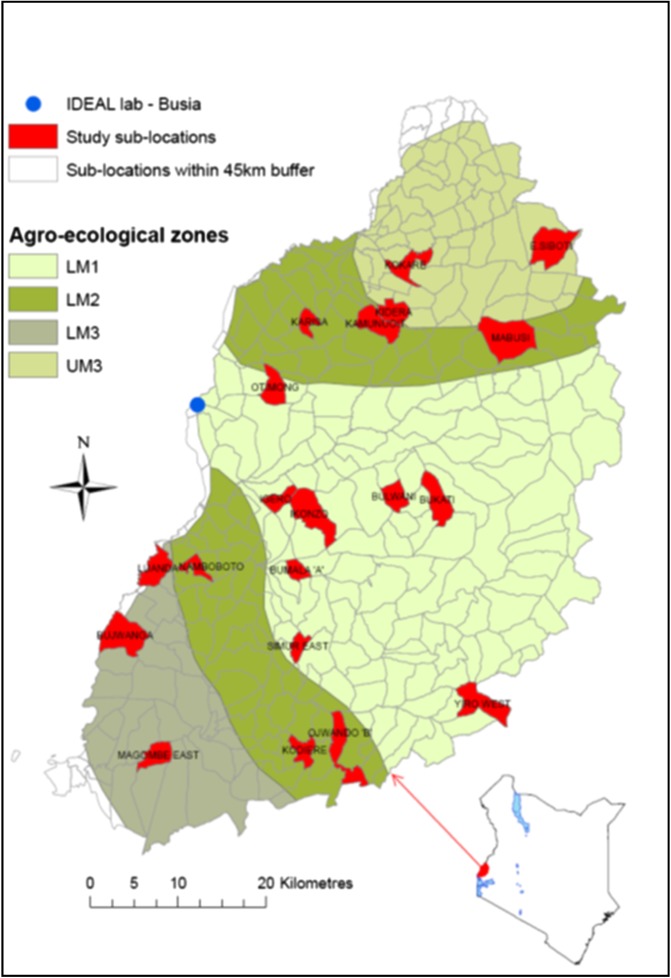
Map of Busia town, Western Kenya showing agroclimatic zones (AEZ) and highlighting the sublocations falling within the 45 km buffer from Busia. The 20 study sublocations included in the study are shown in red.

The study site was in Busia district, western Kenya (Fig. 1). A full description of the study design is given by Bronsvoort *et al*. (2013). The climate is tropical and the main land use in the area is cultivation of maize, sugarcane, cotton, pigeon-peas and sisal. The study focused on the traditional smallholder livestock-keeping farmers who primarily keep indigenous East African Short-horn Zebu cattle. Calves recruited into the study remained with their herd of origin, and were exposed to the natural pathogen challenge in the field. Owners were asked to call the Infectious Diseases of East African Livestock (IDEAL) team if a calf was observed to be ill between visits and one of the project veterinary surgeons would examine the calf and treat it if considered to be seriously ill or if there were welfare concerns. Calves were censored from the project after any visit where a treatment was begun.

A total of 548 calves were recruited between October 2007 and September 2009. Calves were sampled according to a stratified 2-stage clustering design. Stratification was by agro-ecological zone (AEZ) with 1st stage cluster (sub-location – smallest official administration unit) selected using stratified-random sampling with replacement and 2nd stage cluster (calf) selected using ordinary random sampling without replacement. Calves were recruited within 3–7 days of birth and followed routinely at 5-weekly intervals until 51 weeks of age. To avoid the effect of seasonality, recruitment was staggered to include calves born during each month of the recruitment period. In addition to routine visits, calves were visited when a clinical episode was reported by either the farmer or the local animal health technician. No interventions against any pathogen or parasite were carried out during the study period.

This research (project V027-09) was approved by the Research Committee of the Faculty of Veterinary Science and the Animal Use and Care Committee of the University of Pretoria. The IDEAL project (OS 03-06) was approved by the Ethics Review Committee of the College of Medicine and Veterinary Medicine at the University of Edinburgh.

### Sample collection, processing and diagnostics

At each visit a complete clinical examination on the calf was performed by trained animal health technicians or veterinarians and biological samples collected included two thin and two thick peripheral blood smears from the marginal ear vein, blood from the jugular vein in a 10 mL serum vacutainer tube and two 5 mL EDTA plastic tubes for haematology, and fecal samples directly from the rectum of the calf (Bronsvoort *et al*. 2013).

Initial sample processing and analysis occurred at the local field laboratory, Busia, Kenya. Packed cell volume was measured using a Hawksley microhaematocrit reader (Jain, 1993). The WBC and Plt were measured using an automated blood cell analyser (pocH-100iV Diff, Sysmex^©^ Europe GMBH). Thin blood smears were air-dried, fixed in absolute alcohol and stained for 30 min with Giemsa 10% dilution. Thick blood smears were air-dried, and stained as described above. Smears were examined for blood-borne parasites with a standard light microscope under 10–100× magnification. The microhaematrocrit centrifugation and dark-ground/phase-contrast buffy coat techniques (OIE, 2005) were used to identify trypanosomes in blood. The McMasters method (Kaufmann, 1996) was used to calculate the number of nematode eggs (EPG) and number of coccidial oocysts (OPG) per g feces. Nematode eggs were differentiated as either strongyle-type eggs or strongyloides-type eggs. The presence of trematode eggs was determined by sedimentation of feces (Kaufmann, 1996).

Serum samples were tested with indirect enzyme-linked immunosorbent assays (ELISA) to determine the antibodies titres to *T. parva, T. mutans, A. marginale* and *B. bigemina* (Katende *et al.* 1990, 1998; Morzaria *et al.* 1999; Tebele *et al*. 2000). The results were expressed as per cent positivity (PP) values of optical density, relative to those of a strong positive control (Wright *et al*. 1993). A PP cut-off of 20 was considered positive for *T. parva* and *T. mutans*, and a PP cut-off of 15 was considered positive for *A. marginale* and *B. bigemina*.

The age at which seroconversion took place was determined using a moving window rule, relying on three consecutive visits (A, B and C). Calves were classed as seroconverted at visit B if the antibody PP levels was above the species-specific PP cut-off at that visit, the PP was higher than at visit A, and if the PP at visit C was at least 5 PP higher than at visit A.

A multi-species un-nested PCR was used to test for specific trypanosome species (Thumbi *et al*. 2008) on the 51-week samples, as well as samples from individual visits where a calf tested positive for trypanosomes on microscopy.

### Statistical analysis

The computation of the results and the production of the graphs were done with R 2.8.1 (R Development Core Team, 2012).

#### Univariable analysis: The impact of single pathogens

Generalized mixed-effect (GME) models were used in univariable models to investigate the association between pathogens and the haematological measure of interest. The outcome variables considered included PCV, WBC and Plt. Univariable analysis for each independent variable *vs* each haematological measure was used as a preliminary screening tool to determine which independent variables to include in the subsequent multivariable analysis. Univariable models treated the variables representing pathogens as well as age (to account for known age-related physiological changes in dependent variables; Van Wyk *et al.* 2013) as fixed effects, with calf identification (individual) and sublocation as random effects. Consistent inclusion of sublocation conditioned the models for environment and nutrition-related factors and differences in exposure levels to pathogens (Van der Waaij *et al*. 2003). Varying slopes and intercepts models were used and temporal autocorrelation within calves was accounted for in the model specification. Alpha (type 1 error rate) was set at 0.05 for statistical significance.

Pathogens considered for analysis as independent variables included the tick-borne parasites *Theileria* spp., *T. mutans, T. parva, Anaplasma* spp., *A. marginale* and *B. bigemina; Trypanosoma* spp. and specifically *Trypanosoma vivax*; and the intestinal parasites strongyle-type nematodes, strongyloides-type nematodes, coccidia and *Fasciola gigantica*. In some cases pathogen presence/absence was modelled; in others a categorical variable indicating pathogen load was used. Further details follow.

*Theileria* spp. and *Anaplasma* spp. were diagnosed by microscopy and no species differentiation was made. There were only four visits where *Babesia* spp. was detected by microscopy and thus *Babesia* spp. was not included as a covariate.

*Theileria parva, T. mutans, A. marginale* and *B. bigemina* diagnosis was based on seroconversion. The age at seroconversion was used as a covariate in the analysis to capture the acute response around the time of infection. To capture the long-term impact of infection on haematological variables, an indicator for seroconverted status was included as a covariate and for this purpose a calf was considered as positive (seroconverted) for all visits post seroconversion. Because no treatment interventions were carried out during the study and also due to the chronic nature of infections with both helminth and blood-borne pathogens, it can be assumed that a calf remained infected for the remainder of the study period (see Table 1).

**Table 1.** The significance of *P*-values (*P*<0·1) obtained from univariable analysis of single covariates. Age was included as a covariate in all analyses to account for the significant physiological changes in response variables associated with age. Blood-borne pathogens were classed as either present/absent, based on microscopy (mcr) or PCR. For tick-borne diseases age at seroconversion was used as a covariate to capture the acute response around the time of infection. To capture the long-term impact of infection a calf was considered as positive (seroconverted) for all visits post seroconversion. Intestinal parasites were considered as a categorical covariate at three levels, namely negative, positive with a high parasite load or positive with a low parasite load

Covariate		Response variable		
		PCV^a^	WBC^b^	Plt^c^
*Theileria* spp. (mcr)^d^	Presence/absence	****	NS	****
*T. parva*	Positive	NS	**	****
	At seroconversion	**	**	*
*T. mutans*	Positive	****	NS	****
	At seroconversion	***	NS	**
*Anaplasma* spp. (mcr)^d^	Presence/absence	NS	NS	NS
*A. marginale*	Positive	*	NS	****
	At seroconversion	**	NS	**
*B. bigemina*	Positive	NS	***	****
	At seroconversion	NS	NS	**
*Trypanosoma* spp. (mcr)^d^	Presence/absence	****	****	****
*T. vivax* (PCR)^e^	Positive	NS	*	****
Coccidia	Presence/absence	****	***	NS
	OPG^f^>1000	*	NS	NS
Strongyle-type species	Presence/absence	****	NS	****
	EPG^g^>1000^b^	****	****	NS
*Strongyloides* spp.	Presence/absence	NS	**	****
	EPG^g^>1000	NS	NS	*
*Fasciola gigantica*	Presence/absence	NS	NS	NS

****P<0·0001, ***P<0·001, **P<0·01, *P<0·05, NS, non-significant (*P*>0·05).

a PCV: packed cell volume.

b WBC: white blood cell count.

c Plt: platelet count.

d mcr: microscopy. This indicates the test used to diagnose the pathogen.

e PCR: polymerase chain reaction. This indicates the test used to diagnose the pathogen.

f OPG>1000: Oocysts per g feces per >1000.

g EPG>1000: Eggs per g feces >1000.

During initial data exploration, with regards to TBD, the order of infection was included in the analysis, but was found to not significantly affect any of the haematological outcomes statistically, and was thus not included in the final models.

*Trypanosoma-*positive calves were deemed to be those calves that were diagnosed positive by microscopy (mcr) and no distinction between species was made. *Trypanosoma vivax-*positive calves were diagnosed by microscopy and speciation confirmed by PCR.

Strongyle worm species were considered as a categorical covariate at two levels (present or absent) or at three levels, namely negative, positive with a high EPG of strongyle-type eggs (EPG>1000) or positive with a low EPG (EPG<1000). The same classification was used for *Strongyloides* spp. at two levels (present or absent), or at three levels as either negative, or positive with a high EPG (>1000) or low EPG (<1000) of strongyloides-type eggs. Similarly coccidia were analysed as either at two levels or as a three-level categorical covariate as either negative, or based on the OPG, as positive with either high OPG (>1000) or low OPG (<1000). *Fasciola gigantica* was only categorized as either present or absent.

#### Multivariable analysis: the impact of interaction between co-infecting pathogens

Generalized additive mixed models (GAMM) are an extension of GME models and allow the use of the non-parametric smoothers in addition to parametric components where applicable (Wood, 2006). Non-parametric smoothers were used to account for the non-linear effect of age on the distribution of the haematological variables. Modelling of GAMM was done in R using the package *mgcv* (1·7–0). With *mgcv*, generalized cross validation (GCV) criterion or unbiased risk estimator (UBRE) are used to estimate the smoothing parameters (Crawley, 2007). Models are fitted using penalized likelihood maximization (Crawley, 2007).

All covariates and two-way interactions were treated as fixed effects with the exception of individual (calf identification) and sublocation, which were treated as random effects.

The final multivariable model was determined by first including all covariates (pathogens) significant in the univariable analysis then assessing the fit and significance of each two-way interaction term in a step-up process.

The estimated coefficient of each covariate included in the minimal model was interpreted as an increase in the response variable equal to the coefficient when the covariate (pathogen) was positive (two-level categorical covariates), or an increase equal to the value of the coefficient for an increase in one unit of the covariate (for three-level categorical covariates). A negative value of a coefficient was interpreted as a decrease in the response variable, calculated in the same way as for positive coefficients.

To improve model fit, log (log_10_) transformation of Plt data was necessary. The model parameters reported represent the log-transformed data. The coefficient is interpreted as a percentage increase in the response variable equal to the value of the coefficient when positive, or a percentage decrease in the response variable when the coefficient is negative.

Model fit was assessed by graphical inspection of model residuals (not shown). Akaike's information criteria (AIC) were used as the criterion to compare models.

#### Pathogen interactions

Interaction between the co-infecting pathogens is said to occur when the outcome of infection, e.g. a reduction in PCV, during co-infections differs from the sum of the individual outcomes of the single infections. A positive interaction occurs when the outcome of infection is increased compared with the sum of the individual outcomes, e.g. a more severe reduction in PCV than what is expected to occur due to both pathogens combined. A negative interaction occurs when the outcome of infection is reduced compared to the sum of the individual outcomes, e.g. the reduction in PCV is less severe than what is expected from the combined effect of the individual pathogens.

For the purpose of this study, only two-way interactions between pathogens, as well as interactions between pathogen status and age, were considered for inclusion into the models.

#### Predicted outcomes of interactions between co-infecting pathogens

The predicted mean value of the response variables for each model was calculated for uninfected calves, calves positive for each single covariate, as well as two-way combinations between pathogen pairs. The model-predicted mean value was calculated from the sum of the intercept and the coefficients of the selected pathogens included in the minimal model. The 95% confidence intervals (95% CI) for the predicted mean values were calculated in the same way, using the standard error (s.e.) of each coefficient. To calculate the predicted mean Plt (pPlt), the model-predicted log interval (mean±95%CI) was first calculated and then back transformed through exponentiation (10^*x*^) by the calculated value (*x*).

## RESULTS

### Univariable analysis

The covariates tested in univariable analysis are listed in Table 1. The covariates found to significantly predict the various response variables were further used in model-building in multivariable models. The distribution of pathogens within the study population is described by Bronsvoort *et al*. (2013).

The pathogens that showed significant associations (*P*<0·1) with PCV as response variable using the univariable analysis included the blood-borne pathogens *Theileria* spp. (mcr), *T. parva* (only at the time of seroconversion), *T. mutans, A. marginale, Trypanosoma* spp. (mcr); as well as intestinal parasites coccidia and strongyle-type helminths. The pathogens that showed significant associations (*P*<0·1) with WBC using the univariable analysis included *T. parva; B. bigemina* (after seroconversion), *Trypanosoma* spp. (mcr) and *T. vivax*; coccidia, and both strongyle- and strongyloides-type helminths. All the pathogens showed significant associations (*P*<0·1) with Plt (log-transformed data) using univariable analysis except *Anaplasma* spp. (mcr), coccidia, strongyle-type nematodes (at high parasite loads) and *F. gigantica*.

### Generalized additive mixed effect models

#### Packed cell volume

The multivariable GAMM model for PCV showed effect of age on PCV was significant but non-linear and hence was included as a covariate with a smoothing function (estimated degrees of freedom (edf) = 8·79 and *P*<0·0001). Pathogens that caused a significant (*P*<0·05) decrease in PCV include *Theileria* spp. (mcr); *T. mutans; Trypanosoma* spp. (mcr); *A. marginale* and strongyle worms. Strongyle species had a more severe effect on PCV when EPG was high. The only pathogen that caused a significant increase in PCV was coccidia (*P*<0·05). *Theileria mutans* caused a decrease in PCV at the time of seroconversion only, thus in acute infections. *Anaplasma marginale* infection was associated with a decrease in PCV, not only at the time of seroconversion but throughout the remainder of the follow-up as seroconverted calves maintained the deficit in PCV. The coefficient estimates of the GAMM model for PCV are found in Table 2.

**Table 2.** The final generalized mixed-effect model analysis of packed cell volume (*n* = 3917) after exclusion of all statistically non-significant covariates

Covariate	Coeff^a^	s.e.^b^	*P*^c^
*Theileria* spp. (mcr)^d^	−1·064	0·169	<0·0001
*T. mutans* (seroconverted)	−1·222	0·248	<0·0001
*A. marginale* (seroconverted)	−1·583	0·428	<0·001
*Trypanosoma* spp. (mcr)^d^	−5·681	1·012	<0·0001
Strongyle-type species (EPG>1000)^e^	−1·967	0·193	<0·0001
Coccidia (positive)	0·637	0·131	<0·001
*T. parva* (at seroconversion): age	−0·003	0·001	0·043
*A. marginale* : *Theileria* spp.	1·146	0·420	0·006
*T. parva* (seroconverted) : *Theileria* spp.	−0·889	0·279	0·001
*Trypanosoma* spp. : *Theileria* spp.	0·502	0·226	0·03
Strongyle-type nematode : Strongyloides-type nematodes (EPG>1000)	−1·537	0·551	0·005
*B. bigemina* : Coccidia	−1·611	0·691	0·02

a Coefficient.

b Standard error of coefficient.

c *P*-value indicate significance of coefficient.

d mcr (microscopy).

e Eggs per g feces >1000.

a:b indicates interactions between pathogen a and b.

A positive interaction was found between strongyle-type nematodes and strongyloides-type nematodes. The decrease in PCV caused by strongyle infections was 1·78 times more severe in the presence of a concurrent high infectious load of *Strongyloides* spp. infection.

A negative interaction was found between *T. parva* and *Theileria* spp. *Theileria parva* infections caused a very small decrease in PCV at the time of seroconversion, which increased in severity with age of seroconversion. After seroconversion, calves positive for *T. parva* had a significant decrease in PCV (−0·562) only when they were also positive for *Theileria* spp. on microscopy.

A negative interaction was found between *Trypanosoma* spp. and *Theileria* spp. Although both pathogens caused a decrease in PCV by themselves, the total decrease in PCV during co-infections with *Trypanosoma* spp. and *Theileria* spp. (−2·46) was less than single infections with *Trypanosoma* spp. (−5·68).

#### White blood cell count

The effect of age on WBC was significant but non-linear and was included in the model as a covariate with a smoothing function (edf = 1 and *P*<0·0001). The coefficient estimates of the model are found in Table 3. Pathogens that had a negative impact on WBC are strongyle-type nematodes (EPG>1000) and *T. parva* (at the time of seroconversion). The only pathogen that had a significant positive impact on WBC was *Trypanosoma* spp. The magnitude and statistical significance of the effect of *Trypanosoma* spp. on WBC was dependent on the age of the calf.

**Table 3.** The final generalized additive mixed-effect model analysis of mean white blood cell count (*n* = 4680) after exclusion of all statistically non-significant covariates

Covariate	Coeff^a^	s.e.^b^	*P*^c^
Strongyle-type nematodes (EPG>1000)^d^	−0·587	0·101	<0·0001
*Trypanosoma* spp. (mcr)^e^: age	0·006	0·001	<0·001
*Theileria parva* (at seroconversion)	−0·345	0·165	0·03

a Coefficient.

b Standard error of coefficient.

c *P*-value indicate significance of coefficient.

d Eggs per g feces >1000.

e Microscopy.

a:b indicates interactions between pathogen a and b.

#### Platelet counts

The platelet counts were log transformed in all analyses to stabilize the variance and normalize the distribution of the residuals. The model summary estimates are listed in Table 4. The only pathogen that caused a significant increase in Plt was *Strongyloides* spp. Pathogens that caused a significant decrease in Plt included *A. marginale, B. bigemina, Theileria* spp., *T. mutans, T. parva, Trypanosoma* spp. (mcr), *T. vivax* and strongyles. *Trypanosoma* spp. caused the most severe decrease in Plt.

**Table 4.** The final generalized additive mixed-effect model analysis of log-transformed platelet counts (*n* = 3856) after exclusion of all statistically non-significant covariates

Covariate	Coeff^a^	s.e.^b^	*P*^c^
*Anaplasma marginale*	−0·405	0·077	<0·0001
*Babesia bigemina*	−0·092	0·04	0·021
*Theileria* spp.	−0·096	0·033	<0·0001
*Theileria mutans*	−0·117	0·03	<0·001
*Theileria parva*	−0·307	0·076	<0·0001
*Trypanosoma* spp. (mcr)^d^	−1·647	0·326	<0·0001
*Trypanosoma vivax*	−0·37	0·094	<0·0001
*Strongyle* spp.	−0·114	0·04	<0·01
*Strongyloides* spp.	0·122	0·036	<0·001
*Theileria* spp.: *A. marginale*	0·227	0·059	<0·001
*Theileria parva*: *Strongyle* spp.	0·197	0·075	0·009
*Theileria parva*: *Fasciola* spp.	0·142	0·066	0·03
*Theileria mutans*: *Strongyloides* spp.	−0·188	0·058	0·001
*Theileria mutans*: *Trypanosoma* spp.	0·416	0·167	0·013
*Trypanosoma* spp.: *Strongyle* spp.	0·854	0·325	0·009

a Coefficient.

b Standard error of coefficient.

c *P*-value indicate significance of coefficient.

d Microscopy.

a:b indicates interactions between pathogen a and b.

The decrease in Plt was 1·48 times more in *A. marginale*-positive calves than calves positive for both *A. marginale* and *Theileria* spp. The decrease in strongyle-positive animals was twice as low when also positive for *T. parva. Theileria parva* also interacted with *Fasciola* spp. On its own, *Fasciola* spp. had no significant impact on Plt, but in *T. parva*-positive calves a decrease of 16%.

There was interaction between *Trypanosoma* spp. and strongyles. Co-infection with these two pathogens resulted in a decrease in Plt almost eight times more than in strongyle infection alone. An interaction between *Trypanosoma* spp. and *T. mutans* was also detected. The decrease was ten times more than in *T. mutans*-positive calves that were not infected with *Trypanosoma* spp. as well.

### Model-predicted mean of the haematological response variables

#### Predicted packed cell volume

The model-predicted mean PCV (pPCV) at 150 days of age was calculated to illustrate the cumulative impact of blood-borne pathogens (Table 5) and between blood-borne pathogens and intestinal parasites (Table 6) on the PCV of the calves.

**Table 5.** The GAMM-predicted mean packed cell volume (%) (95% confidence intervals) in co-infections with pathogen pairs at 150 days

Covariate	Uninfected	*Theileria* spp. (mcr)	*Theileria mutans* (at seroconversion)	*T. parva* (at seroconversion)	*T. parva* (seroconverted)	*A. marginale* (seroconverted)	*B. bigemina* (seroconverted)	*Trypanosoma* spp. (mcr)
Uninfected	29·33 (28·75–29·92)	–	–	–	–	–	–	–
*Theileria* spp.(mcr)^a^		28·27 (27·69–28·85)	27·05 (26·32–27·77)	27·89 (27·21–28·57)	28·78 (28·14–29·43)	27·85 (27·1–28·61)	28·29 (27·71–28·86)	26·89 (25·44–28·33)
*Theileria mutans* (at seroconversion)		28·11 (27·38–28·85)	27·74 (26·94–28·54)	28·12 (27·38–28·86)	26·52 (25·47–27·57)	28·12 (27·38–28·86)	22·41 (20·31–24·51)
*Theileria parva* (at seroconversion)				29·33 (28·75–29·92)	−	27·75 (26·80–28·70)	29·33 (28·75–29·92)	23·65 (21·61–25·70)
*Theileria parva* (seroconverted)			−	29·95 (28·29–29·62)	27·37 (26·38–28·36)	28·95 (28·29–29·62)	23·27 (21·20–25·34)
*Anaplasma marginale* (seroconverted)					27·75 (26·8–28·7)	27·75 (26·8–28·7)	22·07 (19·90–24·24)
*Trypanosoma* spp. (mcr)^a^							23·64 (21·61–25·70)	23·65 (21·61–25·70)
Strongyle-type nematodes (EPG^b^<1000)		28·39 (27·90–28·88)	28·23 (27·55–28·91)	29·07 (28·48–29·66)	29·45 (28·95–29·95)	27·87 (26·97–28·77)	29·45 (28·95–29·95)	23·77 (21·75–25·80)
Strongyle-type nematodes (EPG^b^>1000)		26·30 (25·63–26·98)	26·14 (25·32–26·97)	26·99 (26·24–27·73)	27·37 (26·69–28·05)	27·78 (24·77–26·80)	27·37 (26·69–28·05)	21·69 (19·61–23·77)
Strongyloides-type nematodes (EPG^b^>1000)		28·27 (27·69–28·85)	28·11 (27·38–28·85)	28·95 (28·29–29·62)	29·33 (28·75–29·92)	27·75 (26·80–28·70)	29·33 (28·75–29·92)	23·65 (21·61–25·70)
Coccidia		28·91 (28·30–29·51)	28·75 (27·99–29·51)	29·59 (28·90–30·28)	29·97 (29·37–30·58)	28·39 (27·42–29·36)	28·36 (26·92–29·81)	24·29 (22·24–26·34)

a mcr: microscopy.

b EPG: eggs per g feces.

**Table 6.** The GAMM-predicted mean white cell blood count (×10^3^ *μ*L^−1^) (95% confidence intervals) at age 150 days for co-infections with pathogen pairs

Covariate	Uninfected	*Trypanosoma* spp.	*Theileria parva*	Strongyle-type nematodes (EPG>1000)
Uninfected	10·75 (10·55–10·95)	–	–	–
*Trypanosoma* spp. (mcr)		11·65 (11·14–12·17)	11·31 (10·71–11·91)	11·07 (10·51–11·63)
*Theileria parva* (at seroconversion)			10·41 (10·05–10·77)	9·82 (9·41–10·24)
Strongyle-type nematodes (EPG>1000)^a^				10·17 (9·87–10·47)

a EPG eggs per g feces.

The pathogen group that caused the most severe decrease in pPCV was *Trypanosoma* spp. (mcr). Strongyle-type helminths caused the second most severe decrease in pPCV, but the decrease was only clinically significant at a high EPG. Other pathogens that caused a decrease in mean pPCV were *T. mutans, T. parva, Theileria* spp. and *A. marginale* but these small decreases were not likely to be clinically significant unless they occurred as part of multi-pathogen infections. Coccidia caused a slightly higher pPCV than in uninfected calves, but this was unlikely to be clinically significant.

**Fig. 2.**
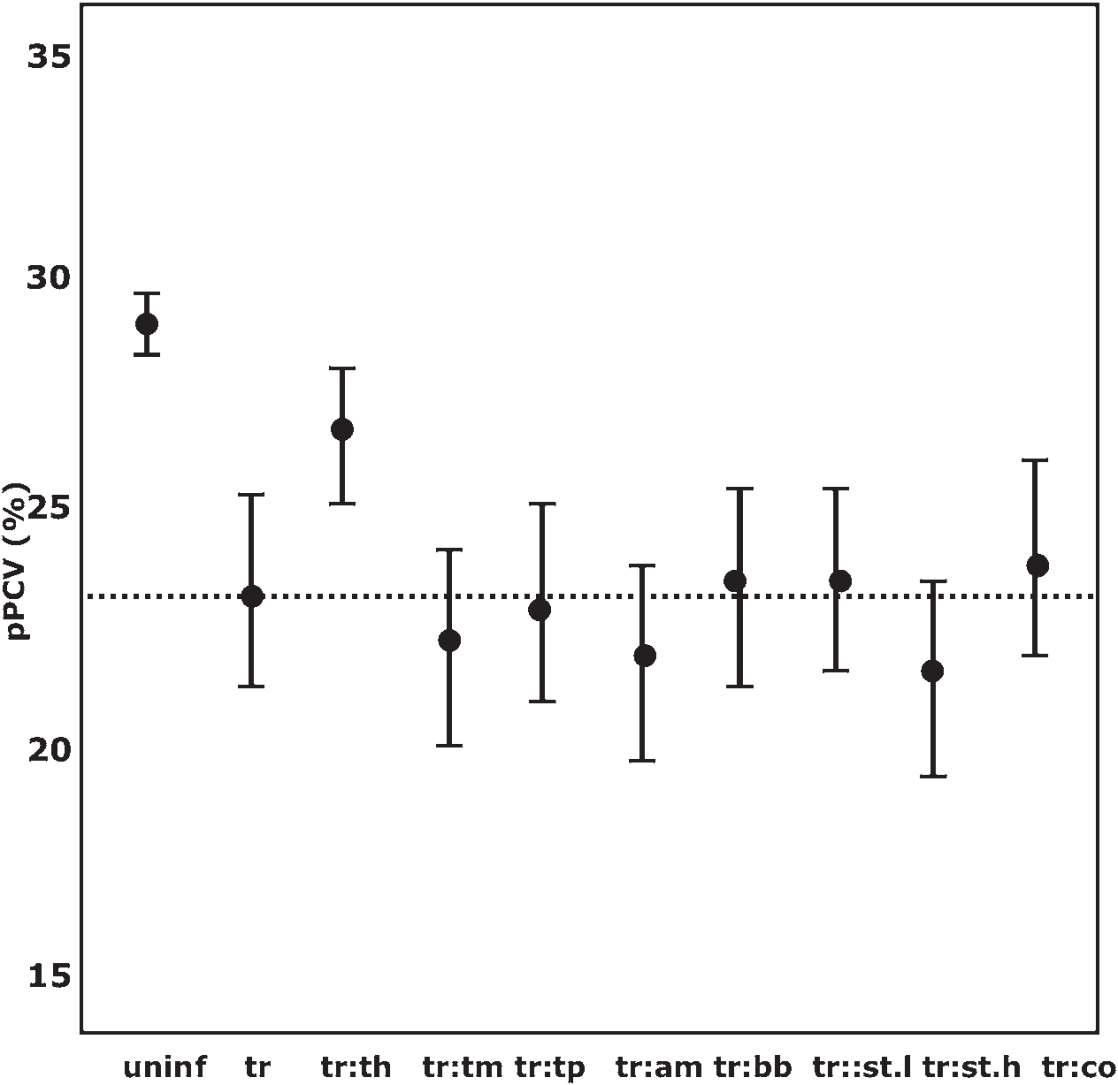
The distribution of the GAMM-predicted mean packed cell volume in co-infections associated with *Trypanosoma* spp.-positive calves at 150 days of age. pPCV, model predicted mean packed cell volume; a:b denotes co-infection between pathogen a and b; uninf, uninfected; tr, *Trypanosoma* spp.; th, *Theileria* spp.; tm, *Theileria mutans*; tp, *Theileria parva*; am, *Anaplasma marginale*; bb, *Babesia bigemina*; st.l, Strongyle-type worms (EPG<1000); st.h, Strongyle-type worms (EPG>1000); ---, mean pPCV of *Trypanosoma* spp.-positive calves.

There was a cumulative decrease in pPCV in co-infections between *Trypanosoma* spp. and strongyle-type helminths, *T. mutans, T. parva* and *A. marginale*. The overall lowest mean pPCV was found in co-infections with *Trypanosoma* spp. and strongyle-type species (EPG>1000) (mean pPCV = 21·69% at 150 days). Co-infection with coccidia only marginally improved the pPCV. The mean pPCV at 150 days in concomitant infections between *Trypanosoma* spp. and other pathogens is illustrated in Fig. 2.

Combinations of tick-borne pathogens also resulted in cumulative decreases in PCV. Co-infections of *T. mutans* and *A. marginale* resulted in a decrease in PCV of almost 3% compared with uninfected calves. Co-infections of *T. mutans* and *Theileria* spp. resulted in a decrease in PCV of 2·3%, and between *T. mutans* and *T. parva* (at seroconversion) of 1·6% compared with uninfected calves.

#### Predicted white blood cell count

The model-predicted mean WBC (pWBC) at 150 days of age was calculated to illustrate the impact of the cumulative effect and interaction between pathogens on WBC. The results are tabulated in Table 6. The pathogen with the most significant impact on pWBC was *Trypanosoma* spp. This pathogen caused an increase of 0·9×10^3^ *μ*L^−1^ in pWBC in calves of 150 days, compared with uninfected calves of the same age. Strongyles caused a decrease in pWBC but only when the EPG>1000. Co-infections between *T. parva* and strongyle-type nematodes resulted in the most significant decrease in pWBC than uninfected calves.

#### Predicted platelet counts

The model-predicted mean platelet counts (pPlt) associated with co-infection with different pathogen pairs was calculated to illustrate the impact of co-infections of pathogens on the Plt of calves (Table 7).

**Table 7.** The back-transformed GAMM-predicted mean platelet counts (×10^3^ *μ*L^−1^) (95% confidence intervals) at age 150 days for co-infections with pathogen pairs

Covariate	Uninfected	*A. marginale*	*B. bigemina*	*Theileria* spp.	*T. mutans*	*T. parva*	*Trypanosoma* spp.	*T. vivax*	Strongyle-type nematodes	*Strongyloides* spp.
Uninfected	1657 (1368–2006)	–	–	–	–	–	–	–	–	–
*Anaplasma marginale*		651 (444–957)	527 (346–805)	882 (625–1245)	648 (447–943)	417 (272–641)	15 (3–67)	278 (160–484)	502 (356–707)	862 (586–1269)
*Babesia bigemina*			1341 (1032–1742)	1076 (827–1400)	1025 (780–1348)	661 (458–954)	30 (6–134)	572 (351–934)	1032 (834–1278)	1775 (1376–2291)
*Theileria* spp.				1329 (1094–1616)	1015 (821–1257)	655 (474–907)	30 (7–130)	567 (356–904)	1023 (907–1155)	1760 (1455–2128)
*Theileria mutans*					1266 (1018–1575)	625 (451–864)	74 (16–347)	540 (337–868)	975 (837–1136)	1088 (827–1430)
*Theileria parva*						817 (589–1133)	18 (4–81)	349 (204–595)	990 (848–1157)	1082 (768–1523)
*Trypanosoma* spp. (microscopy)							37 (8–162)	16 (4–68)	205 (113–375)	49 (11–216)
*Trypanosoma vivax*								707 (445–1125)	544 (351–845)	936 (591–1482)
Strongyle-type nematodes									1275 (1126–1444)	1688 (1421–2006)
*Strongyloides* spp.										2193 (1832–2625)

**Fig. 3.**
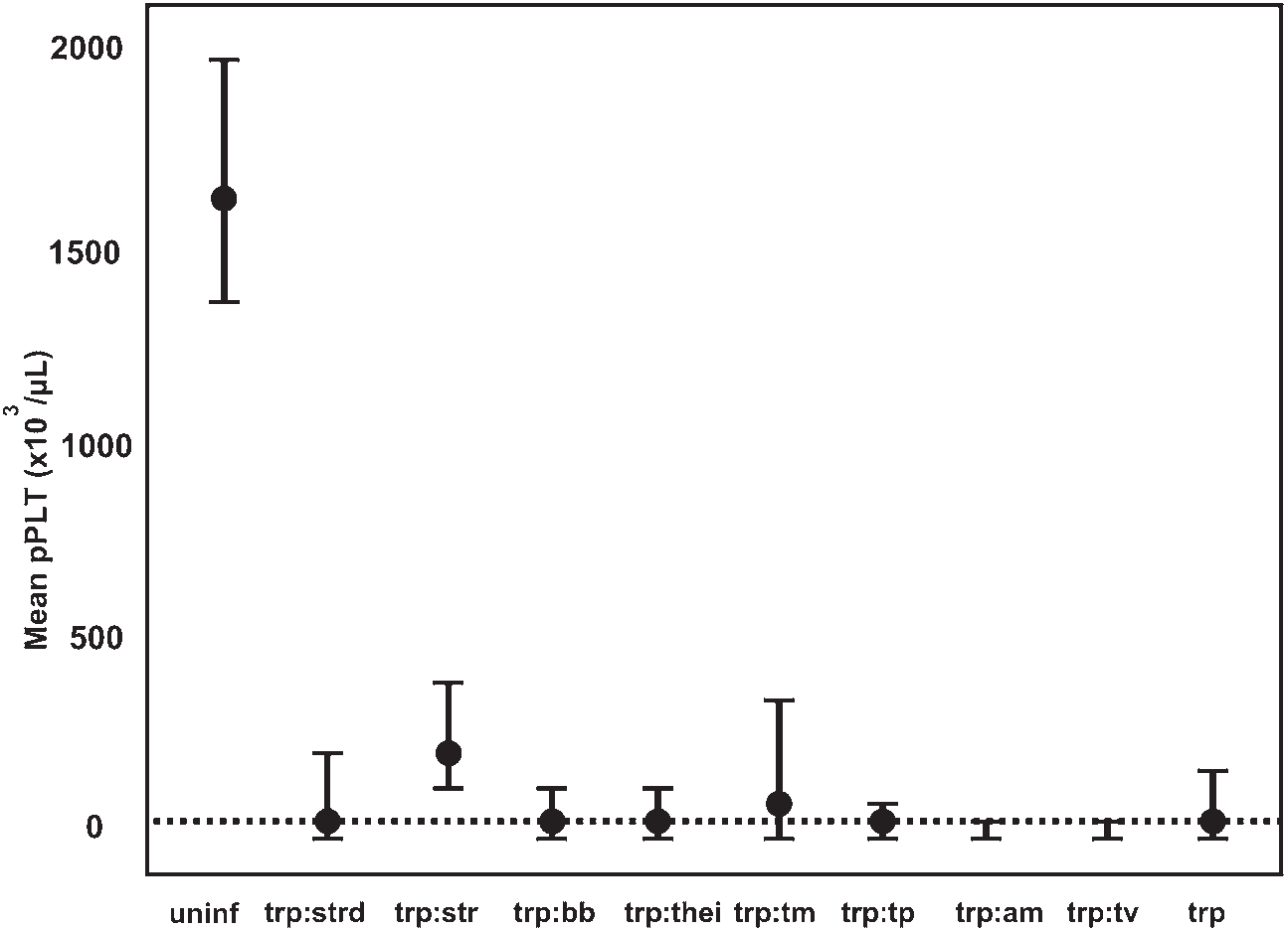
The GAMM-predicted mean platelet counts for co-infections associated with *Trypanosoma* spp. infections. pPLT, model predicted platelet count; a:b denotes co-infection between pathogen a and b; uninf, uninfected; trp, *Trypanosoma* spp.; strd, Strongyloides-type worms; str, Strongyle-type worms; bb, *Babesia bigemina*; thei, *Theileria* spp.; tm, *T. mutans*; tp, *T. parva*; am, *Anaplasma marginale*; tv, *T. vivax*; ---, mean pPLT of *Trypanosoma* spp.-positive calves.

The pathogen that had the most significant impact on pPlt was *Trypanosoma* spp. (mcr). The mean pPlt for *Trypanosoma*-infected calves was less than 3% of the mean in uninfected calves. The mean pPlt in calves that were positive for both *Trypanosoma* spp. (mcr) and *T. vivax* (PCR) was half (16×10^3^ *μ*L^−1^) of the pPlt in calves only positive on microscopy. The distribution of pPlt for various co-infections associated with *Trypanosoma* spp. is illustrated in Fig. 3.

**Fig. 4.**
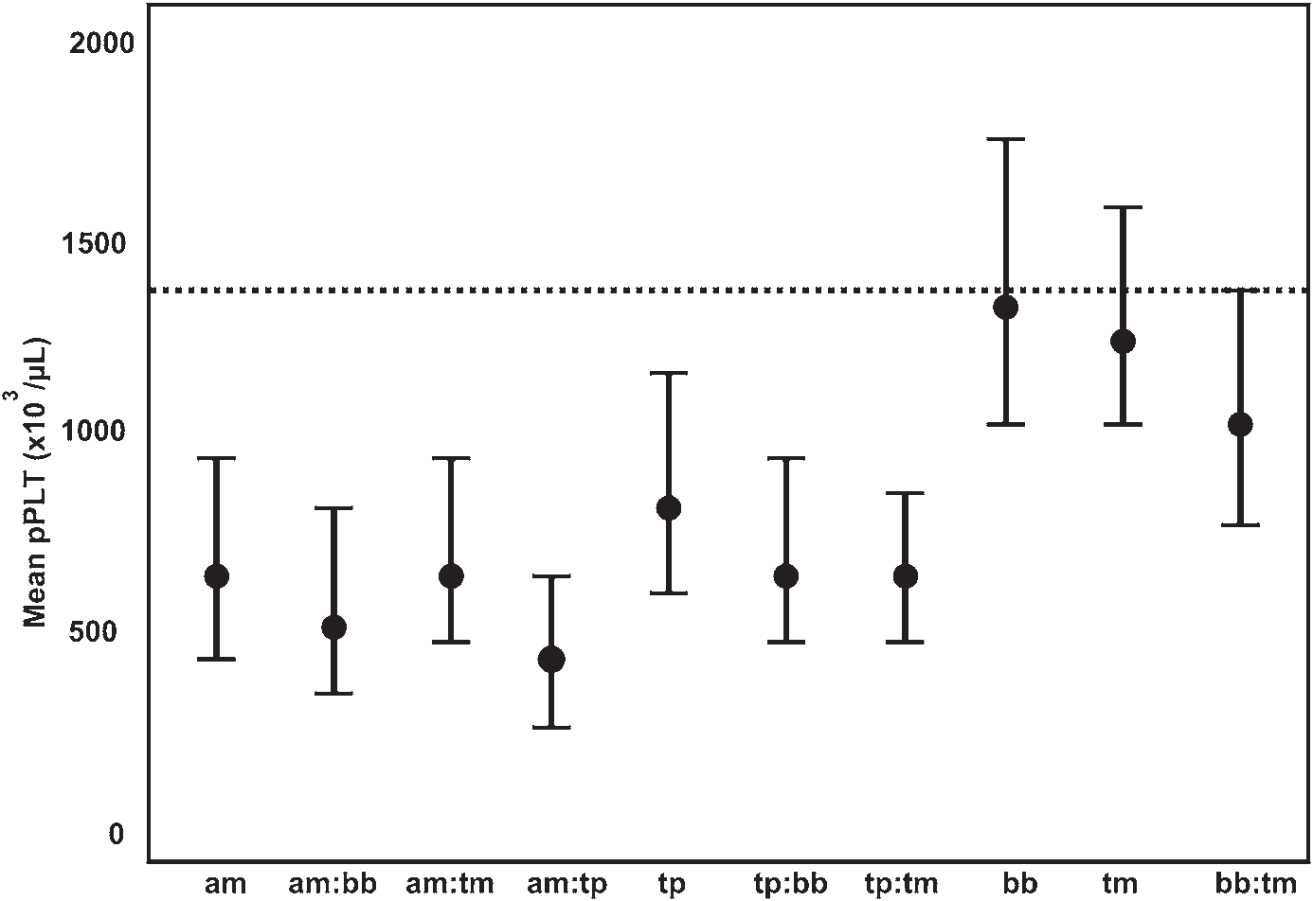
The GAMM-predicted mean platelet counts for co-infections associated with tick-borne pathogens. pPLT, model predicted platelet count; a:b denotes co-infection between pathogen a and b; am, *Anaplasma marginale*; bb, *Babesia bigemina*; tm, *T. mutans*; tp, *T. parva*; ---, mean pPLT of uninfected calves.

Tick-borne diseases all caused a significant drop in pPlt, although not as severe as the trypanosomes. *Anaplasma marginale* had the lowest pPlt of the tick-borne pathogens, with a mean pPlt of 651×10^3^ *μ*L^−1^. The lowest pPlt for tick-borne disease occurred in concomitant infections with *A. marginale* and *T. parva*. Co-infections between *T. parva* and the other tick-borne parasites also resulted in decreased pPlt. The distribution of pPlt for the various co-infections with tick-borne parasites is illustrated in Fig. 4.

The only pathogen that caused an increase in pPlt was *Strongyloides* spp.

## DISCUSSION

### Packed cell volume

*Trypanosoma* spp. was an important pathogenic cause of anaemia in the calf population. Although the pPCV in all co-infections involving *Trypanosoma* spp. were well below the pPCV of uninfected calves, interactions with other pathogens, apart from strongyle-type nematodes, further reduced the final pPCV only marginally compared with single infections with *Trypanosoma* spp. The pathogenicity of *Trypanosoma* spp. partially depends on the intensity of the parasitaemia. The parasitic wave during trypanosome infections coincides with a drop in PCV (Murray and Dexter, 1988). Due to its relatively low sensitivity compared with molecular diagnostic tests, microscopy is more likely to test positive at the peak of such parasitic waves (Uilenberg, 1998), and thus in times of high parasitaemia. This would explain in part why *Trypanosoma* spp. (mcr) is associated with such a significantly reduced PCV. *Trypanosoma vivax*, which was diagnosed by PCR, was not associated with a significant decrease in PCV in this calf population.

Strongyle-type nematodes were the second most important cause of anaemia in the calf population. The pathogenicity of strongyles depended on its infectious load, with a more significant decrease in PCV as the parasitic load (EPG) increased. *Haemonchus*, a strongyle-type nematode, is considered as one of the most pathogenic parasites of ruminants (Kaufmann 1996) and is consistently reported as the most prevalent helminth species in cattle in Kenya (Moll *et al*. 1984; Latif *et al*. 1995; Waruiri *et al*. 2001, 2002). The pathogenesis is that of a haemorrhagic anaemia (Kaufmann *et al*. 1992). During extremely high parasite burdens the animal may die due to haemorrhagic blood loss. In chronic cases animals develop a steady drop in PCV and serum albumin which results in emaciation of the animal. If the animal survives, the compensatory erythropoiesis will eventually deplete iron reserves, which results in a non-regenerative progressive anaemia. Interaction between strongyles and other pathogens, apart from coccidia, also resulted in a reduced PCV compared with single infections with strongyles. Co-infection with strongyle-type nematodes at high EPG and *Trypanosoma* spp. caused the most severe decrease in PCV compared with uninfected animals. The cumulative impact of these two pathogens can potentially result in anaemias severe enough to cause mortality.

Coccidia caused a slight increase in PCV in this population which was not clinically significant and was possibly due to a level of dehydration due to diarrhoea. Although coccidial infections in calves are predominantly non-pathogenic, coccidiosis in young calves can result in haemorrhagic diarrhoea (Kaufmann, 1996). The pathogenicity of coccidia depends on the species involved. Clinical coccidiosis in calves is most commonly caused by *Eimeria zuerni* and *Eimeria bovis* (Kaufmann, 1996). It would be interesting to investigate the association between coccidia and other clinical parameters, such as the presence or absence of diarrhoea, in this population.

The total decrease in PCV caused by *T. mutans* was of interest. *Theileria mutans* is generally considered to be less pathogenic than *T. parva* (Kariuki, 1990). The 1·2% decrease in PCV in *T. mutans*-positive calves compared with uninfected calves probably does not cause overt clinical symptoms in infected calves; it is, however, evidence of subclinical disease processes. The contribution of *T. mutans* to the level of anaemia in the population becomes of more significance with concomitant infections with other pathogens, such as tick-borne diseases and strongyles.

*Theileria parva* also caused a small decrease in PCV at seroconversion which coincided with a decrease in both WBC and Plt. This decrease in PCV at seroconversion increased in severity as the age at seroconversion increased. *Theileria parva* was also associated with a small decrease in PCV after seroconversion but only in calves also positive on microscopy as indicated by a positive *Theileria* spp. status. Calves positive on serology, but negative on microscopy represent calves that either have cleared the infection or are latent carriers in which the parasitaemia is too low to be detected by microscopy (Young *et al*. 1990). The detection of parasitaemia on microscopy is thus indicative of a higher parasitic load which caused the lower PCV.

*Theileria* spp. (mcr) was associated with a significant decrease in PCV in calves that seroconverted to neither *T. mutans* nor *T. parva*. Speciation of *Theileria* spp. was not done on microscopy. One can therefore not rule out that other *Theileria* species was the reason for the decrease in PCV.

Although clinical anaplasmosis and babesiosis in cattle are classically associated with anaemia, neither *A. marginale* nor *B. bigemina* appeared to be significant causes of anaemia in the study population. This implies that the majority of calves infected with either of these pathogens are probably latent carriers. There is evidence of subclinical disease processes in both pathogens, however, and in particular in association with multi-pathogen infections, as seen by a decrease in thrombocytes.

### White blood cell counts

The pathogen with the most significant impact on WBC was *Trypanosoma* spp. Acute trypanosomosis typically causes an initial leukopenia, as part of a total pancytopenia, which coincides with the first parasitaemic wave. This leukopenia is later followed by a leukocytosis (Murray and Dexter, 1988). The long interval between observation points probably does not reflect the acute change in WBC in trypanosome-infected calves in this study, and thus the initial leukopenia was not captured. It was interesting that the pWBC with *Trypanosoma*-positive calves was dependent on the age of the calves. One can speculate whether this can be ascribed to increase in maturity of cellular immunity in older animals.

The pWBC of calves negative for any of the tested pathogens also increased with age. This is probably due to challenge by pathogens other than what is accounted for in the model.

*Theileria parva* caused a decrease in WBC at the time of seroconversion, after which time no significant difference was found in WBC between seroconverted calves and those that had not seroconverted. Chronic ECF is classically associated with a leukopenia (Maxie *et al*. 1982; Irvin, 1983; Lawrence *et al*. 2004). The WBC has been used as an indicator of prognosis in cases of ECF: animals that maintain their WBC during infection are more likely to recover from ECF (Irvin, 1983). Zebu cattle that are raised in ECF-endemic areas exhibit a low innate susceptibility (Perry and Young, 1995), and this is probably reflected in the insignificant difference in WBC between calves that have seroconverted and those that have not.

Interestingly, strongyles were associated with a decrease in WBC. This decrease in WBC was only significant in high intensity infections (EPG>1000). This decrease in WBC, together with the decrease in RBC, is likely due to a loss in whole blood associated with a heavy *Haemonchus* burden, which is a blood feeder.

### Platelet count

Thrombocytopenia is also potentially an important syndrome in the population.

*Trypanosoma* spp. caused the most significant decrease in platelet counts. Co-infections with other pathogens decreased the pPlt even more, although not considerably. The causes of thrombocytopenia in trypanosomosis are multifactorial. The first cause is parasite by-products that cause initial damage to these cells, while immunological reactions, such as antigen-antibody complexes and auto-antibodies to platelets maintain the thrombocytopenia (Murray and Dexter, 1988). The formation of platelet aggregations has been histologically shown in *T. vivax* infections (Murray and Dexter, 1988) and *Trypanosoma rhodesiense* (Davis *et al*. 1974), which indicates a consumptive loss of thrombocytes. These platelet aggregations are thought to be due to antibodies directed against platelets (Assoku and Gardiner, 1989). In extreme cases, fibrin deposits that form due to disseminated intravascular coagulation (DIC) further damage thrombocytes. Platelet counts as low as those observed in infections associated with *Trypanosoma* spp. may cause coagulopathies, and clinically present as generalized petechiation and ecchymosis. This was confirmed by postmortems that were performed on calves that died during the course of this study (results not shown here).

All four tick-borne infections caused a reduction in Plt, particularly *A. marginale* and *T. parva*. Co-infections between all pairs of tick-borne diseases resulted in a cumulative decrease of platelets. The pathogenesis of the reduced Plt in all four infections is multifactorial, and includes a combination of factors from reduced platelet production and increased consumption to immune-mediated platelet destruction (Pantanowitz, 2003). Splenomegaly is a common symptom of many tick-borne diseases, and also contributes to low Plt due to sequestration of platelets and destruction by macrophages in the spleen (Pantanowitz, 2003). There is thus a cumulative reduction in Plt during co-infections with tick-borne diseases.

## CONCLUSION

*Trypanosoma* spp. and strongyle-type nematodes were the two main causes of anaemia in the calves in the study area. Infections with either of these two pathogen types typically result in chronic progressive infections that are very erosive on the productive capacity of a bovine population. Infections with both pathogens are preventable and treatable, however, and also do not necessarily require sophisticated tests to diagnose. If farmers are educated in basic clinical monitoring techniques, such as the colour of mucous membranes of calves, and minimal sample collection, such as fecal samples, they can, with the help of local veterinary officials, develop a basic but sustainable disease control programme for their own herds.

Co-infections between pathogen pairs were shown to have an impact on the haematological profile of infected calves. Clinically, the impact on haematological parameters holds several implications for the host. Firstly, concomitant infections can complicate the clinical presentation, and thus diagnosis of disease. The animal might present with clinical symptoms that cannot be explained by the disease that was diagnosed. Co-infections could lead to a missed diagnosis when, in the case of concomitant infections with pathogens that present with similar clinical signs, the diagnosis was solely based on clinical presentation. Such a host might not respond to treatment as expected and this should prompt the investigator to consider further diagnostic procedures.

Co-infection between pathogens also affects the prognosis of a disease state. Even if the contribution of the pathogen in itself was clinically insignificant, the cumulative effect of the various co-infecting pathogens could potentially shift the host from a state of apparent health into a state of clinical disease. Immunosuppression caused by certain pathogens, such as *Trypanosoma* spp. or *T. parva*, or immune-modulation, as has been described in helminth infections (Maizels and Yazdanbakhsh, 2003), could undermine the host's response against other pathogens which in turn increase the host's susceptibility to infection or impede its ability to resolve such an infection. Premune latent carrier animals have been reported to develop clinical anaplasmosis after super-infection with *T. vivax* and *Trypanosoma congolense* (Magona and Mayende, 2002).

Interactions between pathogens are not limited to pairs of pathogens, and to get a complete understanding of disease processes one would have to consider the whole pathogen neighbourhood of the host as well as additional pathogen-, host- and environmental-related factors. There were many more pathogens circulating in the population, e.g. viral and bacterial diseases that were not taken into consideration in the analysis, but probably affected the haematological profile of these calves. There are several pathogen-related factors that dictate the pathogenicity of the infection and thus the clinical presentation in the host, including infectious load, pathogen strain, and virulence types within strains, which were not investigated either. Several inherent characteristic of the hosts themselves, such as breed and age, would also determine the susceptibility of the animal to infection, whereas other acquired attributes such as maternal antibody, premunity due to prior infection, or nutritional state could have altered the animals’ ability to respond to infection and therefore their haematological response.

The East African Short-horn Zebu breed is well-adapted to the environmental challenges and infectious burdens it faces under field conditions (Perry and Young, 1995). They survive under conditions where other breeds, such as improved European cattle breeds, do not. Despite the adaptive traits and the tolerance of this indigenous breed to the endemic pathogens, infectious disease remains a major cause of production losses in livestock in East Africa (Uilenberg, 1995). It is evident that interactions between concomitant pathogens complicates the clinical outcome of infected calves and should be taken into consideration in any disease management programme. One should bear in mind, however, that there is a fine balance in the stability of the relationship between pathogens, vectors and hosts. Preventive intervention against one pathogen might compromise the endemic stability of other pathogens, which in turn can result in an endemically unstable situation where even the most tolerant livestock breeds will succumb to disease.
